# Recognition of Pulseless Ventricular Tachycardia Through the Second Analysis of Automated External Defibrillators, Leading to Successful Shock Delivery in a Patient With Dilated Cardiomyopathy: A Case Report

**DOI:** 10.7759/cureus.40755

**Published:** 2023-06-21

**Authors:** Yuki Teragawa, Hiroki Teragawa, Yuichi Orita, Chikage Oshita, Makoto Ochi

**Affiliations:** 1 Department of Clinical Education, JR Hiroshima Hospital, Hiroshima, JPN; 2 Department of Cardiovascular Medicine, JR Hiroshima Hospital, Hiroshima, JPN; 3 Department of Artificial Dialysis Surgery, JR Hiroshima Hospital, Hiroshima, JPN

**Keywords:** return of spontaneous circulation (rosc), pulseless ventricular tachycardia, automated external defibrillator, cardiopulmonary resuscitation practice, idiopathic dilated cardiomyopathy

## Abstract

The use of a defibrillator with a monitor is recommended for the shock indication algorithm for in-hospital cardiac arrest; however, it is likely that many medical facilities are still equipped only with automated external defibrillators (AEDs). We experienced a case of dilated cardiomyopathy (DCM) complicated by pulseless ventricular tachycardia (pVT) in which an AED was used, but shock was deemed unnecessary after the first analysis. We believe that this case is suggestive of resuscitating cardiac arrest, for which defibrillation is indicated and reported here. A 65-year-old man who had DCM and diabetic nephropathy was admitted to our institution because of worsening heart failure. In the hospital, he suddenly had syncope and was diagnosed with cardiac arrest. Thereafter, cardiopulmonary resuscitation (CPR) was performed using an AED, and the monitor on the AED showed pVT. The first analysis of the AED announced unnecessary shock delivery. The pads of the AED were pressed firmly against the chest wall while continuous high-quality CPR was administered for two minutes. The second analysis of the AED revealed the necessity of providing shock for shockable rhythm. The patient experienced the return of spontaneous circulation after shock delivery. We were reminded that there are some clinical cases in which AED shock is not indicated for pVT and that even in such cases, it is important to continue high-quality CPR without panicking.

## Introduction

High-quality cardiopulmonary resuscitation (CPR), including the position, speed, depth of chest compressions, and duration of short interruptions, is essential for cardiopulmonary arrest (CPA) [1]. Moreover, safe and quick defibrillation for shockable rhythm is valid [2]. An automated external defibrillator (AED) is recommended outside hospitals, and a defibrillator with a monitor is recommended in hospitals [2].

Even in our hospital, there are some wards where defibrillators with monitors are not permanently installed, but AEDs are permanently installed. However, there have been many hospitals where only AEDs can be used for shockable rhythm. We encountered a case of dilated cardiomyopathy (DCM) in which the first rhythm was nonshockable when we used an AED for pulseless ventricular tachycardia (pVT). Then, the second rhythm was shockable. Soon after, the patient experienced the return of spontaneous circulation (ROSC). We believe that this is a suggestive case and reported it here.

A partial summary of this case can be found in Preprint (January 2023, DOI: 10.22541/au.167419289.94053314/v1).

## Case presentation

A 65-year-old man who had DCM and underwent peritoneal dialysis (PD), which was introduced due to diabetic nephropathy, was admitted to the hospital because of weight gain and difficulty breathing during light exercise. He reported difficulty breathing during light exercise and gaining weight for three weeks. He had DCM and diabetes. He had no obvious family history of the disease. The patient’s height was 160 cm, and his weight was 81 kg. His blood pressure was 168/91 mmHg, and his heart rate was 71 bpm. His SpO_2_ was 95% (RA), and his body temperature was 36.5°C. His third heart sound was auscultated, the jugular vein was dilated, and edema was observed at the lower extremity.

**Figure 1:**
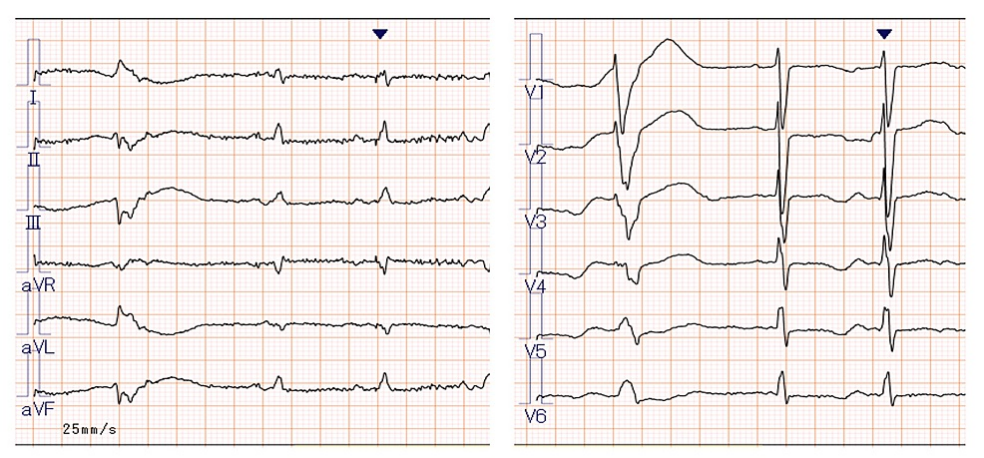
ECG at admission showing premature ventricular contraction

The blood test result showed hypokalemia with 2.9 mEq/L. Electrocardiography showed sinus rhythm with ventricular extrasystole (premature ventricular contraction as shown in Figure 1).

**Figure 2:**
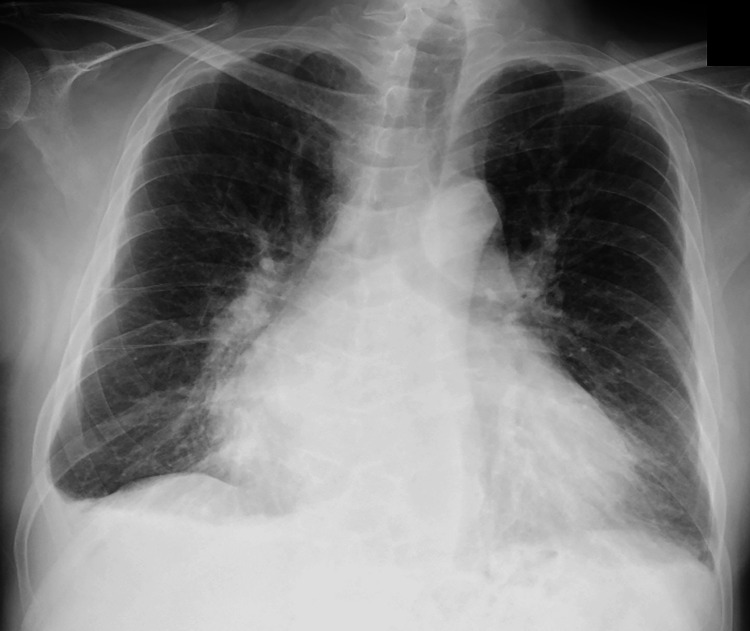
Chest X-ray on admission Chest radiography showed a cardiothoracic ratio of 70% and bilateral pleural effusion.

Chest radiography showed a cardiothoracic ratio of 70% and bilateral pleural effusion (Figure 2).

**Figure 3:**
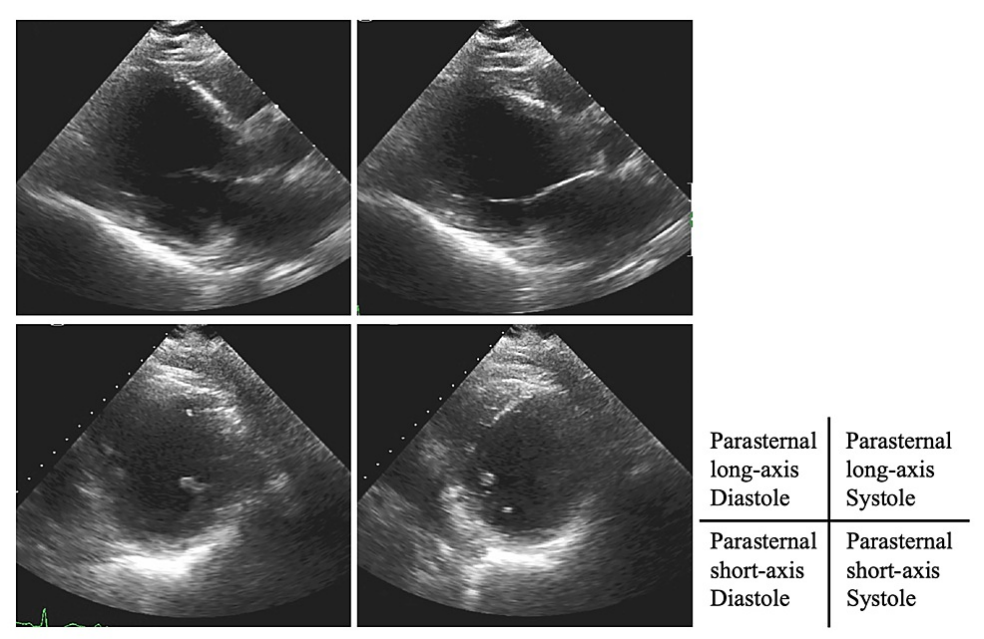
Echocardiography on admission Echocardiography showed a reduced ejection fraction of 19% and left ventricular dilation.

Echocardiography showed a reduced ejection fraction of 19% and left ventricular dilation (Figure 3).

**Figure 4:**
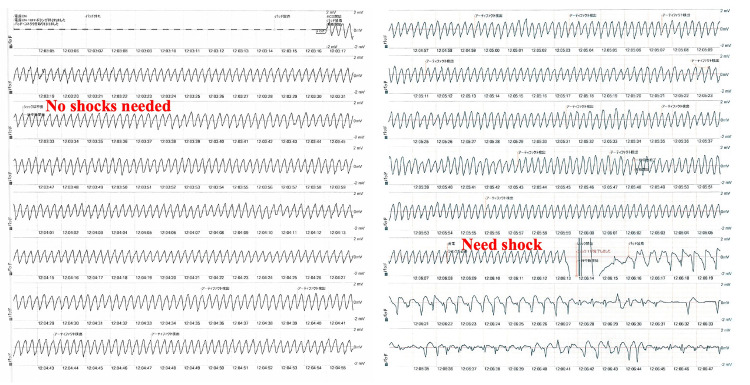
ECG monitor during CPR in the present case After the AED was applied, the first analysis showed that no shock was needed despite the presence of pVT. CPR was continued for two minutes, and after the second analysis showed the need for shock for pVT, shock was delivered. After the success of shock delivery, the patient achieved ROSC. AED: Automated external defibrillator; CPR: Cardiopulmonary resuscitation; ECG: Electrocardiogram; pVT: Pulseless ventricular tachycardia; ROSC: Return of spontaneous circulation.

Four days after admission, the patient was treated with diet therapy, and his body weight did not change on the fifth day. On the fifth day after admission, when we performed an ultrasonographic test of the peripheral blood vessels at the bedside, the patient suddenly lost consciousness. We found that this patient had CPA. Initially, we started CPR with an AED, and the AED monitor showed pulseless VT (Figure 4). However, the first analysis of the AED showed that the delivery of shock was unnecessary, and we had to continue CPR for two minutes. We pressed the AED pads more tightly to the chest wall for two minutes. The second analysis of the AED after two minutes showed that it was necessary to provide shock for the pVT, and we provided shock (Figure 4). The patient experienced ROSC.

**Figure 5:**
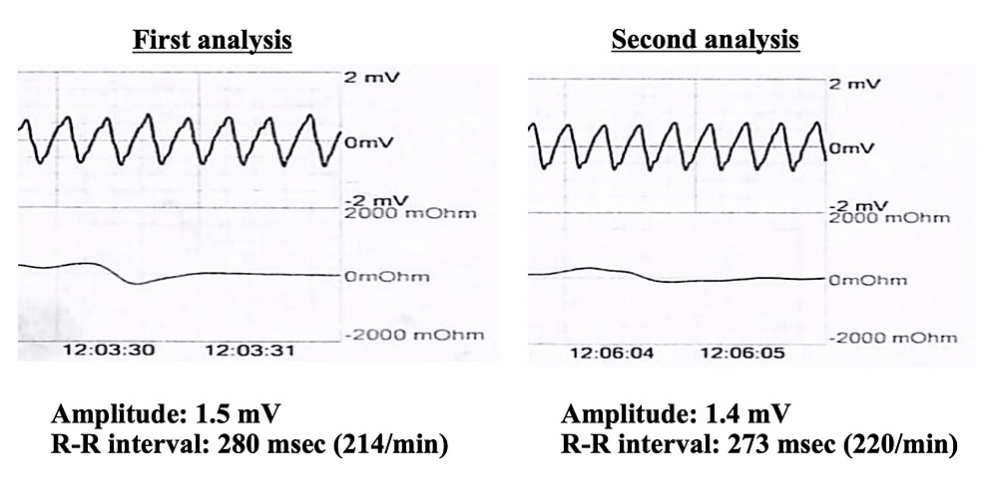
pVT waveforms in the first and second analyses of the AED These figures show that the amplitude of VT was almost unchanged, but the heart rate mildly increased from 214/min to 220/min. AED: Automated external defibrillator; pVT: Pulseless ventricular tachycardia; VT: Ventricular tachycardia.

The pVT values recorded in the first and second analyses are shown in Figure 5, suggesting that there were no significant differences in the amplitude or period of the two pVT waveforms. From the fifth to ninth days, the patient received amiodarone, compensated potassium, and drew water by continuous hemodiafiltration or extracorporeal ultrafiltration. On the 15th day, the patient underwent PD again. On the 35th day, the patient was transferred to another hospital for implantation of an implantable cardioverter defibrillator (ICD).

## Discussion

We encountered a DCM case in which an AED was used for pulseless VT, and ROSC was achieved after the first analysis when no shock was needed and the second analysis when a shock was needed. We considered the cause of pVT to be the worsening of heart failure and hypokalemia. We could not find any morphological differences in the appearance of the first and second pVT waveforms. Moreover, we performed high-quality CPR and pressed the AED pads firmly between CPR compressions, which had some positive effects.

In many emergency cases, the use of AEDs for pVT is recommended as soon as safely possible, and we may overestimate the effectiveness of AEDs for pVT [3]. Generally, the indication for shock by AEDs is influenced by heart rate, waveform, conductivity, and stability, although it may vary depending on the model of AED [4]. In the AED analysis of the present case, the patient’s heart rate during the first and second pVT occurrences was 214/min and 220/min, respectively, which were within the range of the heart rate for shock. However, the frequency of AED shocks for pVT with heart rates of 200-220/min was 5%-15% [3-7]. Through this case, we have to better understand that pVT with such a heart rate may not be recognized as an indication for shock by the AED.

We would like to discuss some measures taken for CPA with pVT in hospitals. First, as recommended in guidelines [3], defibrillators with monitors should be installed in each ward. However, there may be some difficulties due to the financial constraints of the installation. In hospitals that have a defibrillator with a monitor, such as ours, it may be essential to designate a person in charge to take care of the defibrillator with a monitor in case of emergency. After we encountered this case, we revised the hospital manual to clearly state this. Second, some AEDs can be set to defibrillate manually, but the one used at our hospital did not have this function. Even if they have such a function, it is necessary to know how to operate it safely, which may be a problem. Finally, this time, during the first two minutes of CPR, we had to press the AED firmly against the chest wall. This method is recommended in training sessions when the AED pad does not recognize the patient due to excessive chest hair. Although the patient did not have a lot of chest hair, it is possible that the AED pad did not adhere well to the skin due to cold sweat during the shock, so pressing it firmly against the chest wall may have been an indication for shock at the time of the second analysis. It is difficult to determine its effectiveness exactly because it is a single case report, but it may be worth trying. Future verification by a multicenter registry is necessary. Recently, higher defibrillation rates have been reported with AED pads tautly positioned on the chest wall and back, with varying vector positions [8]. It is unknown whether shifting the vector position increases the recognition rate of pVT as an indication for shock, which has to be clarified in future case series. It goes without saying that high-quality chest compressions are important during CPR.

In patients with nonischemic cardiomyopathy and reduced left ventricular ejection fraction, such as the present case, the indication for ICD is class IIa, even if non-sustained VT is not detected [9]. Needless to say, it is important to properly manage electrolytes in such patients and to provide appropriate guideline-based treatment, which we will reflect on and apply in future treatments.

## Conclusions

We encountered a case of DCM in which an AED was used for pVT, and ROSC was achieved after providing shock to the patient in the first analysis when no shock was needed and in the second analysis when a shock was needed. However, AEDs sometimes do not work properly for patients with pVT. So, it is recommended that a defibrillator with a monitor be installed or prepared instantly in each ward. Even if a defibrillator with a monitor is not ready, we should perform high-quality CPR. At that time, strongly pressing AED pads to the chest wall may have a positive effect.
